# Chronic undernutrition (stunting) is detrimental to academic performance among primary schools of adolescent children: a randomized cross sectional survey in Southern Ethiopia

**DOI:** 10.1186/s13104-019-4160-0

**Published:** 2019-03-15

**Authors:** Tsedeke Wolde, Tefera Belachew

**Affiliations:** Population & Family Health Department, College of Health Sciences, Jimma University, P.O. Box 378, Jimma, Ethiopia

**Keywords:** Academic performance, Schoolchildren, Stunting, Southern Ethiopia

## Abstract

**Objective:**

Despite several decades of work in improving childhood nutrition in Ethiopia, stunting remains a major public health concern with lack of evidence on its effect on school performance. Therefore, this study aimed at determining prevalence of stunting and the impact on academic performance among schoolchildren attending primary schools in Meskan District of Southern Ethiopia.

**Results:**

During October 2016, we interviewed primary school children and their parents, collected anthropometric measurements of children, and conducted school record reviews among 408 randomly selected children attending ten primary schools. Univariate and multivariable linear regression were performed to assess stunting associated with academic performance among schoolchildren. A total of 378 primary schoolchildren were involved in the study giving a response rate of 93%. The prevalence of stunting was 16.9%. Grade repetition, place of residence, class rank of students and absenteeism from the class was negatively associated with the academic performance. In general, the present study children had relatively high prevalence of chronic undernutrition. We found that school underperformance was significantly high in the study area. The study underlines the need for nutrition interventions targeting rural areas to improve children’s academic performance.

## Introduction

Poor academic achievement has been a major area of concern for educators, parents, and school children for more than three decades. There are several reasons for children to underperform at school such as age at enrollment, absenteeism, concentration in the classroom, psychosocial factors, illness and nutritional problems. Nutritional problems are among major factors that affect learning and school performance [1–3]. Some local studies conducted in Ethiopia revealed the relationship between academic performance and stunting. A study from Southwest Ethiopia found a significant correlation between stunting and academic performance [4]. Another study from Northwest Ethiopia showed that the low level of educational performance was significantly higher among the stunted children than that of the normal children [5].

Over the past decade, Ethiopia has shown encouraging progress in reducing undernutrition. However, as local studies conducted in Ethiopia revealed the baseline levels of chronic undernutrition (stunting) still remain high. According to the Ethiopia National Nutrition Program (NNP) baseline survey conducted in 2010, 23% of adolescent girls were stunted [6]. Study conducted in West Ethiopia indicates that prevalence of stunting was 17% [7]. A cross sectional study in Eastern Ethiopia shows that the prevalence of stunting was 25.5% [8]. In Northeast Ethiopia, the overall prevalence of stunting among the adolescents (10–19 years) was 15.5% [9]. A school based cross-sectional study in Northwest Ethiopia had revealed the overall prevalence of stunting among adolescent girls was 33.1% [10]. Furthermore, the prevalence of stunting among adolescents was 28.5% [11].

Although chronic undernutrition is a public health problem in Ethiopia, there is little documented information on the nutritional status of schoolchildren and the impact on their academic performance in Southern Ethiopia. Recently, the Ethiopian Ministry of Education recognized the impact of poor health and nutrition on children’s ability to learn, their school attendance, and concentration [12]. The national gross enrolment rate (GER) in primary school (grade 5–8) was 71.1% and 74.2% in Southern Ethiopia. The national repetition rate among primary schoolchildren was 6.7%, slightly higher in males (7.1%) than females (6.2%). The national dropout rate was 10.1% [13]. This study aimed to determine rates of chronic undernutrition (stunting) and correlation with academic performance among primary school children in Meskan District, Southern Ethiopia.

## Main text

### Methods

#### Study design, period and setting

A school-based randomized cross-sectional survey was employed from October to November, 2016 in primary schools of Meskan District in Southern Ethiopia, a food insecure district. Up to 150 undernutrition cases are reported monthly from all health facilities of the District [14]. According to the 2015 population projection of central statistics authorities (CSA), the total population of the Meskan District is 411,933, of these 204,037 are males (CSA, 2013) [15]. Farming is the main occupation. In the 2015–2016 academic terms, 6745 schoolchildren were attending grades 5 to 8.

#### Population and sampling

The source population included all primary school children in Meskan District, with children from randomly selected primary schools constituting the study population. Primary school children, who did not intend to transfer schools during the intervention period, were included. Exclusion criteria included; schoolchildren with obvious congenital or physical growth measurements. We excluded schoolchildren whose parents did not provide written consent or were unwilling to participate.

The sample size was calculated using StatCalc in Epi Info 7. The following parameters used to determine the sample size: 23% of adolescents were stunted [6], 95% confidence level, 4% margin of error and adding 5% for nonresponse, the final sample size was 408 schoolchildren.

Ten primary schools were selected from the forty primary schools using proportional to size (PS) allocation method. Number and list of students were obtained from school. From each selected schools study participants were selected by simple random sampling based on sampling frame existing in the schools (students’ roster).

#### Measurements

A structured questionnaire was developed by reviewing relevant literature, such as the nutrition baseline survey report for the National Nutrition Program of Ethiopia by Ethiopian Public Health Institute (EPHI) [6]. The questionnaire was first prepared in English, then translated into the local language (Amharic), and back translated into English by language experts to check for consistency. The questionnaires were administered in Amharic. The questionnaire was tested on 20 schoolchildren (not included in the sample) selected from primary school in Butajira town, and refined on the basis of the feedback obtained from the pre-test.

Height was measured in an erect position using a calibrated wooden height-measuring board (Stadiometer, Shorr Productions, Olney, MD, USA) with a sliding head bar while children were barefoot. Their height was measured in duplicate and recorded to the nearest 0.1 cm or in triplicate whenever the deviation between the first two measurements was > 0.5 cm and the average of all measurements was taken. The age of children in completed years was obtained from student’s records and confirmed from their parents. In this study, stunting was defined as height-for-age z-score (HAZ) of equal to or less than minus two standard deviation (− 2 SD) below the mean of a reference standard [16].

The overall subjects the students were given in the academic year 2015–2016 were considered to evaluate the academic performance of the students. Annual average grade score was evaluated by taking the result of two consecutive semesters of the year. To assess the relationship of chronic undernutrition (stunting) on academic performance, average marks of the overall subjects the students received were divided into two categories, poor scores and good scores, based on a cut-off mark of 50%. This cut off point was determined by considering the pass mark set by federal ministry of education, Ethiopian [13].

#### Study variables

The outcome variable in the study was academic performance, operationalized as students’ average marks of the overall subjects. Stunting in children was considered as an independent variable in this study. Durable household assets, home animals, housing conditions and vehicles were considered in the construction of household relative wealth index (RWI) using a principal component analysis after checking all the assumptions. RWI divided and ranked into quintile (lowest, second, middle, fourth and highest).

#### Data management and statistical analysis

The data were entered in double, cleaned and checked for missing values and outliers, and analyzed using IBM SPSS statistics for windows, version 23.0. The Z score value for height-for-age was calculated using the WHO AnthroPlus software [17].

Descriptive analyses were performed using frequencies and percentages for categorical variables. Variables which were significant at p-value < 0.2 in the bivariate linear regression model analyses were candidate for entering into the multivariable linear regression model to identify the independent predictors for academic performance. A p < 0.05 was considered statistically significant. We present the results of the linear regression as parameter estimates (ß), p-values and 95% confidence intervals.

### Results

We identified 408 primary school children in the study; however, 30 schoolchildren (17 boys and 13 girls) declined participation, leaving 378 (93%) schoolchildren paired with their parents from ten primary schools who were enrolled in our study. The study children (n = 378) had girl to boy ratio of 1.21:1. The mean age of the children was 12.8 years (SD ± 1.3) with a range of 10 to 15 years. Two hundred two (53.4%) of the schoolchildren were attending grade 6 and while a few students 30 (7.9%) were attending grade 8. Majority of the study participants 273 (72.2%) were from families who were currently lived in rural areas. Two hundred five (54.2%) of them had fathers who attended primary school. Two hundred twenty eight (60.3%) of enrolled students had fathers who were employed as farmers.

One hundred five (27.8%) respondents were categorized in the fourth wealth quintile (Table 1).

**Table 1:** Socio-demographic characteristics, nutritional status and academic performance of participants at primary schools of Meskan District 2016

Variables	Categories	Frequency	Percent
Sex	Boy	171	45.2
	Girl	207	54.8
Grade level	Grade 5	62	16.4
	Grade 6	202	53.4
	Grade 7	84	22.2
	Grade 8	30	7.9
Age in years	10–12	159	42.1
	13–15	219	57.9
Place of residence	Rural	273	72.2
	Urban	105	27.8
Father’s educational status	Illiterate	125	33.1
	Primary complete	205	54.2
	Secondary complete	41	10.8
	Above secondary	7	1.9
Mother’s educational status	Illiterate	189	50
	Primary complete	167	44.2
	Secondary complete	18	4.8
	Above secondary	4	1.1
Father’s occupation	Farmer	228	60.3
	Government employee	28	7.4
	Merchant	71	18.8
	Other	51	13.5
Mother’s occupation	Household work	235	62.2
	Government employee	9	2.4
	Farmer	16	4.2
	Merchant	103	27.2
	Other	15	4
Wealth quintile	Lowest	76	20.1
	Second	72	19
	Middle	61	16.1
	Fourth	105	27.8
	Highest	64	16.9
Family size	< 7	172	45.5
	≥ 7	206	54.5
Grade repetition	Yes	42	11.1
	No	336	88.9
Reason of grade repetition	Academic	4	9.5
	Diseases	10	23.8
	Shortage of food	28	66.7
Stunted children	Yes	64	16.9
	No	314	83.1
Average grade score	< 50%	257	68
	≥ 50%	121	32

In the academic year 2015–2016; 42 (11.1%) students repeated a grade due food shortages of which 28 (66.7%) were in their households. The overall prevalence of stunting was 16.9% (95% CI 13–20.6%). The prevalence of stunting was significantly higher in boys (18.7%) than girls (15.5%). The average score for among children was 64.52 (SD ± 8.45) and the majority (n = 257, 68%, 95% CI 63.5–73%) scored poorly (Table 1).

The majority of the schoolchildren 357 (94.4%) reported that they have good relationships with their peers, and 369 (97.6%) had good relationships with their families. Two hundred thirty nine (63.2%) schoolchildren reported being bullied by one of their families and 233 (61.6%) reported being punished at school. A majority of the parents 303 (80.2%) reported that they have to perform non-school work at their home (Table 2).

**Table 2:** Psychosocial conditions of schoolchildren and parental involvement in schooling at primary schools of Meskan District 2016

Variables	Categories	Frequency	Percent
Good relation with peers	Yes	357	94.4
	No	21	5.6
Good relation with families	Yes	369	97.6
	No	9	2.4
Ever been bulled by families	Yes	239	63.2
	No	139	36.8
Punished at school	Yes	233	61.6
	No	145	38.4
Work load at home	Yes	75	19.8
	No	303	80.2
Family quarrel (at home)	Yes	112	29.6
	No	266	70.4
Their argument disturbed	Yes	72	64.3
	No	40	35.7
Schooling support of the parents			
Regularly attend parent–teacher meeting	Yes	325	86
	No	53	14
Talk about schooling with children	Yes	345	91.3
	No	33	8.7
Support the children during home work	Yes	131	34.7
	No	247	65.3

Three hundred twenty five (86%) of the parents reported that they regularly attend the school meeting (parent–teacher meeting). The majority (n = 345, 91.3%) of the parents said that they always discuss their children schooling with students while 34.9% of the parents support their children in doing their homework (Table 2).

In bivariate linear regression (Table 3), grade repetition and dietary diversity score of students were positively associated with average grade scores, while residence in rural, class rank of students and absenteeism from the class were negatively associated with the average grade scores. After adjusting for height for age z-score (HAZ) and all other variables in the multivariable linear regression model (Table 3), grade repetition, residence in rural areas, student class rank and class absenteeism were negatively associated with the average grade scores.

**Table 3:** Predictors of academic performance of grade at primary schools of Meskan District 2016

Variables	Univariate linear regression				Multivariable linear regression			
	β	95% CI		p-value	β	95% CI		p-value
Age in years	0.070	− 0.206	1.145	0.173				
Sex	0.054	− 0.795	2.638	0.292				
Place of residence	− 0.262	− 6.775	− 3.088	0.000	− 0.119	− 3.541	− 0.927	0.001
Household size	0.076	− 0.108	0.761	0.141				
HH wealth index score	0.068	− 0.283	1.427	0.189				
Iodized salt Level	0.060	− 0.274	1.061	0.247				
Dietary diversity score	0.130	0.135	1.055	0.011	0.025	− 0.196	0.422	0.473
Food frequency	− 0.041	− 0.240	0.102	0.428				
Class rank	− 0.738	− 0.419	− 0.348	0.000	− 0.820	− 0.470	− 0.381	0.000
Grade repetition	0.349	6.805	11.909	0.000	− 0.236	− 8.977	− 3.699	0.000
Absenteeism (class)	− 0.245	− 1.728	− 0.739	0.000	− 0.111	− 0.975	− 0.138	0.009
Height in cm	0.045	− 0.053	0.137	0.388				
Stunting (HAZ)	0.004	− 0.712	0.769	0.940	− 0.055	− 0.892	0.094	0.112

### Discussion

The overall prevalence of stunting in this study was 16.9%, similar to a study conducted in West Ethiopia (17%) [7] and Southwest Ethiopia (16%) [18]. But higher than Addis Ababa (7.2%) [19], East Ethiopia (8.9%) [20], Adama Ethiopia (12.6%) [21], Gonder (12.9%) [22] and Northeast Ethiopia (15.5%) [9]. The possible reason for this discrepancy might stem from differential dietary intake, socioeconomic and cultural differences rather than differences in their genetic potential to achieve maximum height. However, the prevalence of stunting among schoolchildren was lower than National study (23%) [6], Tigray Ethiopia (26.5%) [23], Northern Ethiopia 28.5% [11], and East west Ethiopia (24%) [24].

The current study revealed underperformance when compared with a study conducted in Uganda and India of the primary school children was achieved better academic performance [25, 26]. This lower underperformance in the study schools may be attributed to the poor course delivery system and curriculum. The finding of current study also indicated poor academic performance than that study conducted in Southwest Ethiopia had scored better academic performance [4].

The finding of this study showed that grade repetition, class rank and absenteeism were associated with academic performance of students. Consistent with this study finding, a study done in Ethiopia, India and Malaysia, chronic undernutrition is associated with grade repetition [4, 5, 26, 27]. The finding showed that place of residence was negatively associated with the academic performance, similar in other parts of Ethiopia, Sub-Saharan Africa and Western Africa [4, 28–34].

### Conclusion

In general, the present study children had relatively high prevalence of chronic undernutrition. We found that school underperformance was significantly high in the study area. But, chronic undernutrition was not associated with poor academic performance of grade. The majority children were from subsistence farming households and consume foods grown in their local area. In study settings, the production of foods are mainly using for cash crops. However, there is ample evidence that children’s academic performance outcome can be affected by other factors that were not captured in this study, including micronutrient deficiencies, lack of food availability in their households, medical problems, below average intelligence, specific learning disabilities, attention deficit hyperactivity disorder, emotional problems, a poor sociocultural home environment, psychiatric disorders, or environmental factors, and parasitic infections. The study underlines the need for nutrition interventions targeting rural areas to improve children’s academic performance of grade.

## Limitations

- This study used only anthropomorphic measurements and did not assess the micronutrient status of study participants.
- There may have been differences in the evaluation system for schoolchildren academic performance among the study schools.
- Furthermore, physical performance capacity and motor skills were not measured in this study.

## Abbreviations

HFZ: height for age Z score
MOH: Ministry of Health
NNP: National Nutrition Program
SD: standard deviation
SPSS: statistical package for social science
WHO: World Health Organization

## References

[1] Emerick L (1992). Academic underachievement among the gifted: students’ perceptions of factors that reverse the pattern. Gift Child Q.

[2] Karande S, Kulkarni M (2005). Poor school performance. Indian J Pediatr.

[3] Levinger B. Nutrition health and education, current issue and trends: international basic education programs. 1992.

[4] Abebe F, Geleto A, Sena L, Hailu C (2017). Predictors of academic performance with due focus on undernutrition among students attending primary schools of Hawa Gelan district, Southwest Ethiopia: a school based cross sectional study. BMC Nutr.

[5] Asmare B, Taddele M, B S, Wagnew F (2018). Nutritional status and correlation with academic performance among primary school children, northwest Ethiopia. BMC Res Notes.

[6] National Nutrition Baseline Survey for the National Nutrition Program of Ethiopia. Addis Ababa: Ethiopian Health and Nutrition Research Institute; 2009/10.

[7] Bidu KT, Hailemariam T, Negeri EL (2018). Prevalence and associated factors of undernutrition among school adolescents in Gobu Seyo District, West Ethiopia. J Public Health Epidemiol.

[8] Yebyo HG, Birhane K, Gesesew HA (2015). Assessment of adolescents’ under nutrition level among school students in Eastern Tigray, Ethiopia. J Nutr Food Sci.

[9] Woday A, Menber Y, Tsegaye D (2018). Prevalence of and associated factors of stunting among adolescents in Tehuledere District, North East Ethiopia. J Clin Cell Immunol..

[10] Birru SM, Tariku A, Belew AK (2018). Improved dietary diversity of school adolscent girls in the context of urban Northwest Ethiopia. Italian J Pediatr.

[11] Melaku YA, Zello GA, Gill TK, Adams RJ, Shi Z. Prevalence and factors associated with stunting and thinness among adolescent students in Northern Ethiopia : a comparison to World Health Organization standards. Arch Public Health. 2015; 1–11. 10.1186/s13690-015-0093-9. PMC4624644 26516456

[12] Ministry of Education (2012). National school health and nutrition strategy.

[13] Ethiopian Ministry of Education. Education statistics annual abstract. 2015/2016. http://www.moe.gov.et/English/Resources/Documents/eab05.pdf. Accessed 15 Mar 2017.

[14] Health department of Meskan district (2016). Maternal and Child Nutrition Unit HMIS report of 2016.

[15] Federal Democratic Republic of Ethiopia Population Census Commission. Summary and statistical report of the central statics agency 2013 population and housing census. Addis Ababa: United Nations Population Fund (UNFPA). 2014.

[16] WHO Multicentre Growth Reference Study Group. WHO Child Growth Standards: length/height-for-age, weight-for-age, weight-for-length, weight-for-height and body mass index-for-age: methods and development; 2006. http://www.who.int/childgrowth/standards/Technical_report.pdf. Accessed 10 Mar 2017.

[17] World Health Organization (WHO). WHO AnthroPlus for personal computers manual: software for assessing growth of the world’s children and adolescents. Geneva: WHO; 2009. http://www.who.int/growthref/tools/en/. Accessed 15 July 2016.

[18] Assefa H, Belachew T, Negash L. Socioeconomic factors associated with underweight and stunting among adolescents of Jimma Zone, South West Ethiopia: a cross-sectional study. ISRN Public Health. 2013:7. 10.1155/2013/238546.

[19] Gebreyohannes Y, Shiferaw S, Demtsu B, Bugssa G (2014). Nutritional status of adolescents in selected government and private secondary schools of Addis Ababa, Ethiopia. Int J Nutr Food Sci..

[20] Firehiwot M, Alemayehu W, Yemane B (2015). Prevalence and associated factors of stunting among primary school children in Eastern Ethiopia. Nutr Diet Suppl.

[21] Reji P, Belay G, Erko B, Legesse M, Belay M (2011). Intestinal parasitic infections and malnutrition amongst first cycle primary school in Adama, Ethiopia. Afr J Prim Health Care Fam Med..

[22] Amare B (2012). Nutritional status and dietary intake of urban residents in Gondar, Northwest Ethiopia. BMC Public Health..

[23] Mulugeta A, Hagos F, Stoecker B, Kruseman G, Linderhof V, Zenebe A (2009). Nutritional status of adolescent girls from rural communities of Tigray, Northern Ethiopia. Ethiop J Health Dev..

[24] Hararge B, Çağındaki O, Beslenme A (2015). Nutritional status and associated factors among school adolescent in Chiro Town, West Hararge, Ethiopia. Gaziantep Med J.

[25] Acham H, Kikafunda JK, Malde MK, Oldewage-Theron WH, Egal AA (2012). Breakfast, midday meals and academic achievement in rural primary schools in Uganda: implications for education and school health policy. Food Nutr Res..

[26] Rashmi MR, Shweta BM, Randell S (2015). Prevalence of malnutrition and relationship with scholastic performance among primary and secondary schoolchildren in two select private schools in Bangalore rural district (India). Indian J Community Med..

[27] Shariff ZM, Bond JT, Johnson NE (2000). Nutrition and educational achievement of urban primary schoolchildren in Malaysia. Asia Pac J Clin Nutr..

[28] Mesert Y, Jemal H, Hailu K, Fleming L (2010). Socioeconomic and demographic factors affecting body mass index of adolescents students aged 10–19 in Ambo, Ethiopia. Int J Biol Sci..

[29] Daboné C, Delisle HF, Receveur O (2011). Poor nutritional status of schoolchildren in urban and peri-urban areas of Ouagadougou (Burkina Faso). Nutr J.

[30] Oninla SO, Owa JA, Onayade AA, Taiwo O (2007). Comparative study of nutritional status of urban and rural Nigerian schoolchildren. J Trop Pediatr.

[31] Teller H, Yimar G (2000). Levels and determinants of malnutrition in adolescent and adult women in Southern Ethiopia. Ethiop J Health Dev..

[32] Woldemariam G, Genebo T. Determinants of nutritional status of women and children in Ethiopia, Ethiopia. Addis Ababa, Calverton: Health and Nutrition Research Institute. ORC Macro. 2002.

[33] Zerihun T, Larson CP, Hanley JA (1997). Anthropometric status of Oromo women of child bearing age in rural southwestern Ethiopia. Ethiop J Health Dev..

[34] Duong MC, Mora-Plazas M, Marín C, Villamor E (2015). Vitamin B-12 deficiency in children is associated with grade repetition and school absenteeism, independent of folate, iron, zinc, or vitamin A status biomarkers. J Nutr.

